# Computer-Aided Segmentation and Machine Learning of Integrated Clinical and Diffusion-Weighted Imaging Parameters for Predicting Lymph Node Metastasis in Endometrial Cancer

**DOI:** 10.3390/cancers13061406

**Published:** 2021-03-19

**Authors:** Lan-Yan Yang, Tiing Yee Siow, Yu-Chun Lin, Ren-Chin Wu, Hsin-Ying Lu, Hsin-Ju Chiang, Chih-Yi Ho, Yu-Ting Huang, Yen-Ling Huang, Yu-Bin Pan, Angel Chao, Chyong-Huey Lai, Gigin Lin

**Affiliations:** 1 Clinical Trial Center, Chang Gung Memorial Hospital at Linkou and Chang Gung University, 5 Fuhsing St., Guishan, Taoyuan 33382, Taiwan; lyyang@cgmh.org.tw (L.-Y.Y.); jacky1145@cgmh.org.tw (Y.-B.P.); 2 Department of Medical Imaging and Intervention, Chang Gung Memorial Hospital at Linkou and Chang Gung University, 5 Fuhsing St., Guishan, Taoyuan 33382, Taiwan; tiingyee@cgmh.org.tw (T.Y.S.); jack805@cgmh.org.tw (Y.-C.L.); hsinyinglu@cgmh.org.tw (H.-Y.L.); hsinju0414@cgmh.org.tw (H.-J.C.); silvia0707@cgmh.org.tw (C.-Y.H.); b9102091@cgmh.org.tw (Y.-L.H.); 3 Clinical Metabolomics Core Laboratory, Chang Gung Memorial Hospital at Linkou, 5 Fuhsing St., Guishan, Taoyuan 33382, Taiwan; m7131@adm.cgmh.org.tw; 4 Department of Pathology, Chang Gung Memorial Hospital at Linkou and Chang Gung University, 5 Fuhsing St., Guishan, Taoyuan 33382, Taiwan; qby@cgmh.org.tw; 5 Department of Diagnostic Radiology, Chang Gung Memorial Hospital at Keelung, 222, Maijin Rd., Keelung 20401, Taiwan; 6 Department of Obstetrics and Gynecology and Gynecologic Cancer Research Center, Chang Gung Memorial Hospital at Linkou and Chang Gung University, 5 Fuhsing St., Guishan, Taoyuan 33382, Taiwan; angel945@cgmh.org.tw (A.C.); laich46@cgmh.org.tw (C.-H.L.)

**Keywords:** computer-aided, diffusion-weighted imaging, endometrial cancer, lymph node, metastasis, machine learning, magnetic resonance, radiomics

## Abstract

**Simple Summary:**

Computer-aided segmentation and machine learning added values of clinical parameters and diffusion-weighted imaging radiomics for predicting nodal metastasis in endometrial cancer, with a diagnostic performance superior to criteria based on lymph node size or apparent diffusion coefficient.

**Abstract:**

Precise risk stratification in lymphadenectomy is important for patients with endometrial cancer (EC), to balance the therapeutic benefit against the operation-related morbidity and mortality. We aimed to investigate added values of computer-aided segmentation and machine learning based on clinical parameters and diffusion-weighted imaging radiomics for predicting lymph node (LN) metastasis in EC. This prospective observational study included 236 women with EC (mean age ± standard deviation, 51.2 ± 11.6 years) who underwent magnetic resonance (MR) imaging before surgery during July 2010–July 2018, randomly split into training (*n* = 165) and test sets (*n* = 71). A decision-tree model was constructed based on mean apparent diffusion coefficient (ADC) value of the tumor (cutoff, 1.1 × 10^−3^ mm^2^/s), skewness of the relative ADC value (cutoff, 1.2), short-axis diameter of LN (cutoff, 1.7 mm) and skewness ADC value of the LN (cutoff, 7.2 × 10^−2^), as well as tumor grade (1 vs. 2 and 3), and clinical tumor size (cutoff, 20 mm). The sensitivity and specificity of the model were 94% and 80% for the training set and 86%, 78% for the independent testing set, respectively. The areas under the receiver operating characteristics curve (AUCs) of the decision-tree was 0.85—significantly higher than the mean ADC model (AUC = 0.54) and LN short-axis diameter criteria (AUC = 0.62) (both *p* < 0.0001). We concluded that a combination of clinical and MR radiomics generates a prediction model for LN metastasis in EC, with diagnostic performance surpassing the conventional ADC and size criteria.

## 1. Introduction

Endometrial cancer (EC) is one of the most common gynecological malignancies worldwide. Its incidence rate has increased in successive generations in countries with rapid socioeconomic transitions [1]. Early-stage EC has favorable outcomes [2]; however, the prognosis for patients with lymph node (LN) involvement is considerably poorer. A lymphadenectomy is valuable in defining nodal status and tailoring adjuvant therapy [2]. However, routine lymphadenectomy in patients with EC remains controversial [3,4] because of the potential postoperative morbidity and the technical difficulty of the procedure in obese patients. However, emerging evidence suggests the survival benefit of systematic lymphadenectomy in patients with EC with intermediate or high risk for nodal metastasis [5]. This evidence highlights the importance of precise risk stratification in lymphadenectomy to balance the therapeutic benefit against perioperative morbidity and mortality.

Magnetic resonance (MR) imaging is useful in defining the extent of nodal disease to guide the anatomic border for lymphadenectomy [6]. However, conventional MR imaging using a short-axis diameter of 10 mm or greater to identify suspicious LN could only achieve a modest sensitivity of 48% [7]. Diffusion-weighted (DW) imaging has proven to increase the conspicuity in pelvic LN identification [8,9], but the role of the apparent diffusion coefficient (ADC) values in the prediction of LN metastasis in EC remains debatable. The mean [10] and relative [11] ADC values can be considerably lower in metastatic nodes than in benign nodes, but contradictory results have also been reported [9]. The discordant results in literature may be partly explained by the considerable variations in interobserver and intraobserver reproducibility in measuring LN ADC values [12]. Obtaining reliable ADC quantification for LN is challenging because of the small size of LN. To optimize the diagnostic performance of DW imaging in LN staging, the analytical technique should be refined. To achieve reproducible segmentation results, whole tumor volumetric segmentation, rather than focused selected tumor region of interest (ROI), could be used. LN was segmented using a computer-assisted method based on objective imaging characteristics. The high-throughput radiomic ADC features through machine learning have potentials in building a prediction model to serve as a risk stratification tool for lymphadenectomy and guide the extent of operation through the localization of potential LN metastasis regions.

The aim of this study was to investigate added values of computer-aided segmentation and machine learning based on clinical parameters and diffusion-weighted imaging radiomics for predicting nodal metastasis in endometrial cancer.

## 2. Materials and Methods

### 2.1. Patients and Imaging Protocol

**Figure 1:**
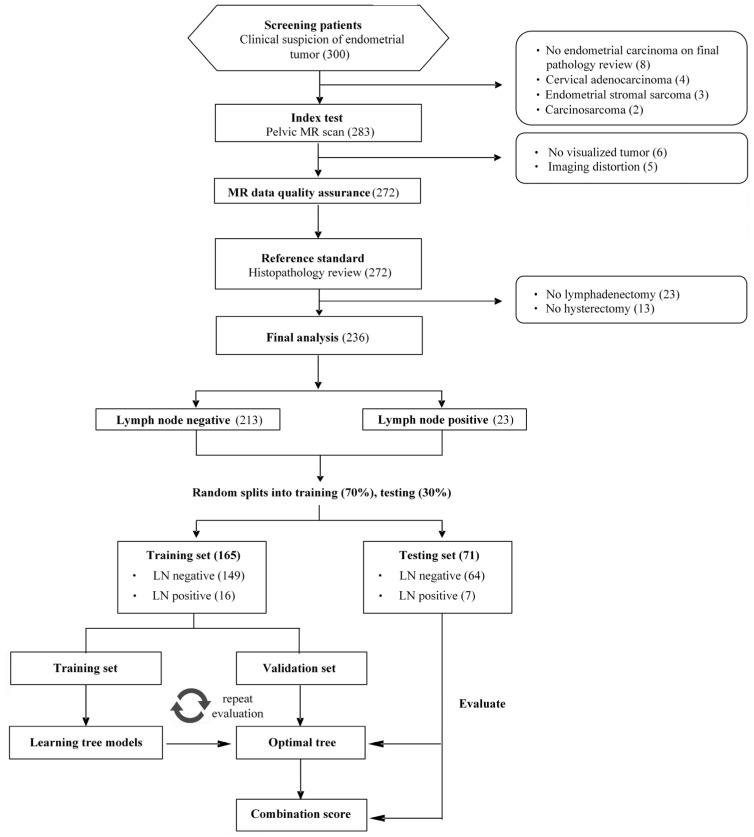
Flowchart of study population. TP = true positive, TN = true negative, FP = false positive, FN = false negative.

This study was performed in a prospective observational cohort diagnosed as having EC during July 2010–July 2018 and during in a tertiary referral center by a dedicated gynecologic oncology interdisciplinary team. The study was approved by the local institutional review board (approval number: IRB101-2187B and IRB103-7316A3), and written informed consent was obtained from all patients. Inclusion criteria were (1) histologically proven and untreated EC for which operations were scheduled and (2) age ≥ 18 years. Exclusion criteria were (1) MR contraindications (cardiac pacemaker, insulin pump, cochlear implant, and metal shrapnel), (2) presence of pelvic or hip metal prostheses, (3) impaired renal function with estimated glomerular filtration rates < 60 mL/min/1.73 m^2^, and (4) inability to provide informed consent. A flow diagram of the cohort selection is presented in Figure 1. All imaging exams were conducted with a 3-T MR scanner (Tim Trio; Siemens, Erlangen, Germany) before the patients were scheduled for operations, with the detailed imaging protocol stated in the Appendix A.

### 2.2. Image Processing and Feature Extraction

**Figure 2:**
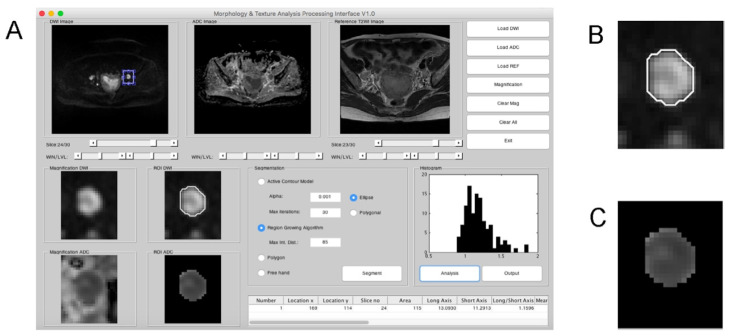
Computer-assisted segmentation of the lymph node (LN) based on diffusion-weighted (DW) imaging. (**A**) The graphical user interface using region growing algorithm for segmentation of LN. The apparent diffusion coefficient (ADC) values of the lymph node are used for histogram analysis. Short and long axes of the segmented lymph node were calculated automatically. (**B**) The region-growing algorithm correctly segmented the LN on DW imaging. (**C**) The segmented ADC map based on the result of (B).

By using in-house developed software written in MATLAB (version 8.3 R2014a; MathWorks, Natick, MA, USA), we manually contoured the ROIs of the main tumors based on DW imaging. Regional largest LNs were segmented using a computer-aided method (Figure 2), and the details are described in the Appendix A. To improve the reliability of ADC comparison, normalized ADC (nADC) was computed. Four classes of ADC parameter were extracted: tumor ADC (ADCt), LN ADC (ADCln), absolute ADC difference between LN and tumor (rADC), and absolute ADC difference between tumor mean value and LN histogram value (rmADC). Each class comprised 12 histogram-derived data: mean, minimum and maximal pixel ADC (ADC_mean_, ADC_min_ and ADC_max_, respectively); 10th-, 25th-, 50th-, 75th-, and 90th-percentile pixel ADC (ADC_p10_, ADC_p25_, ADC_p50_, ADC_p75_, and ADC_p90_, respectively), skewness, kurtosis, standard deviation, and variation. The 48 parameters were normalized with bladder ADC to generate 96 ADC-related parameters. In addition, 6 MR imaging anatomical parameters (tumor volume, and LN: area, long-axis, short-axis, mean diameters, and short-to-long axis ratio) and 7 standard-of-care preoperatively available clinical parameters, including histology [6,13,14], tumor grade [13,14,15,16,17,18,19,20,21], tumor size [18,20,22], low segment location [13,22], presence of deep myometrial invasion [6,13,14,16,18,19,20,21,22], pelvic LN metastasis based on MR report [6], and serum CA-125 level [6,13,15,16,17], were included for analysis. Finally, 109 clinical and radiomic parameters were used for model development.

### 2.3. Histopathology

The reference standard is based on final histopathology. All patients underwent a standard surgical procedure. Surgeons with prior knowledge of the MR imaging findings carefully identified any possible metastasis during pelvic lymphadenectomy. The details are described in the Appendix A.

### 2.4. Statistical Analysis

Descriptive statistics were used to summarize the characteristics of the study population. We used the *t*-test on normally distributed variables, Mann–Whitney U test for non-parametric continuous data, and Chi-square or Fisher’s exact test on categorical data, when appropriate. A weighted decision-tree model based on the classification and regression tree method was applied to build the prediction model for LN metastasis through the training/validation and testing process. The dataset of total 236 patients was randomly split into training and testing sets consisting of 16 LN metastasis and 149 absent patients (70%), and 7 LN metastasis and 64 absent patients (30%) respectively. A decision-tree method on the region-based training data was employed for feature selection and determining the cut-off for the most appropriate model—RadScore, initially including all the MR parameters. The rpart [23] package in R was used, to fit the trees with default cp = 0.01 and setting minsplit = 5 and maxdepth = 4 to control the size of the trees, and 10-fold cross-validation process repeated 10 times was perform to select the best fitting. Thus, a binary RadScore indicated the corresponding classification according to the tree rule can be obtained, and it was then combined with clinical parameters to fit a composite tree model—RadSignature. The success criteria for prediction were set to achieve high sensitivity and negative predictive value (NPV) while maintaining non-inferior specificity to the standard of care based on metrics in internal validation, and the performance of the model was assessed independently using testing set after training/validation step. The quality metrics (sensitivity, specificity, and diagnostic accuracy) of the tree model and conventional single parameter model based on ADC values (ADC model) or LN short-axis diameter (SA model) were determined and presented with 95% confidence intervals. The cut-off values for the ADC or SA models were chosen based on the Youden index. The areas under the receiver operating characteristic (ROC) curve (AUCs) were calculated to compare the diagnostic performance among models based on the De Long methods. All data were analyzed using the SPSS (version 11; SPSS, Chicago, IL, USA), MedCalc for Windows (version 9.2.0.0; MedCalc Software; Mariakerke, Belgium), or R (version 3.4.1). All tests were two-sided, and *p* < 0.05 was considered statistically significant.

## 3. Results

### 3.1. Demographics

From July 2010 to July 2018, a consecutive cohort of 300 patients was enrolled, and a total of 236 patients were eligible for final analysis with mean ± standard deviation age 51.2 ± 11.6 years. Table 1 lists the clinical and demographic characteristics of the study population. The interval between the MR examination and surgery was 27 ± 4 days.

### 3.2. Data Distribution

**Figure A1:**
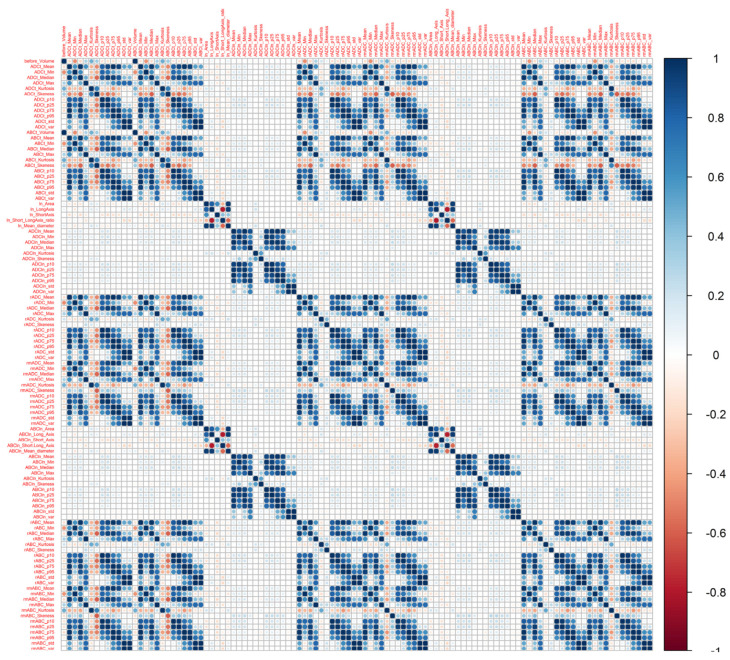
The correlation matrix demonstrated a high correlation among the ADC parameters

An average of 27 nodes per patient was harvested from the pelvic sidewalls (range: 0–83, total: 5078). The positive cases were 33 among the 472 analyzed regions (7.0%), and 23 among the 236 patients (9.7%) based on the final pathology report, suggesting sufficient positive and negative classes for model fitting. The 23 patients with pelvic LN metastasis exhibited significant differences in age, histology, tumor grade, tumor size, deep myometrial invasion and low segment involvement of the uterus, as summarized in Table 1. Patients with metastatic nodes tended to have an older (*p* = 0.004), non-endometrioid type (*p* < 0.001), grade 3 tumor (*p* < 0.001), larger tumor size (*p* = 0.009), deep myometrial invasion (*p* < 0.0001) and low segment involvement on MR imaging (*p* = 0.002). The stepwise multivariate analysis identified the non-endometrioid type and presence of deep myometrial invasion being the independent clinical risk factors. We also found the positive LNs having a significantly larger short-axis diameter (*p* < 0.0001) and short-to-long axis ratio (*p* < 0.0001), and significant lower tumor ADC_mean_ (*p* < 0.0001), ADC_min_ (*p* = 0.003), but not tumor ADC_max_ (*p* = 0.316). The ADC values of the metastatic LNs were significantly lower than those of the benign LNs (ADC_mean_, *p =* 0.049; ADC_min_*, p =* 0.017). The correlation matrix demonstrated a high correlation among the ADC parameters (Appendix Figure A1). None of the LNs showed lobulated or spiculated margins indicating metastasis.

### 3.3. Model Comparison and Subgroup Analysis

**Figure 3:**
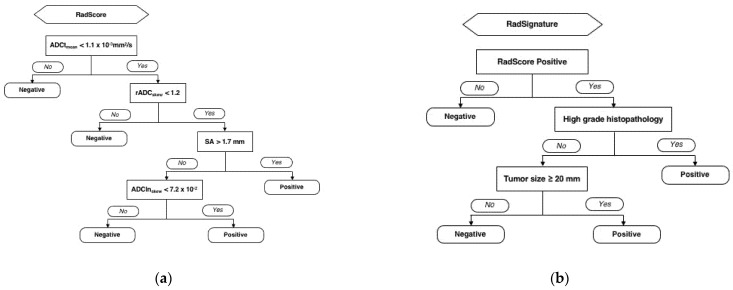
The development of RadSignature to predict lymph node metastasis in endometrial cancer patients. The RadScore was developed based on image only radiomics classification and regression tree analysis (**a**) The RadSignature was determined by combination of RadScore and clinical parameters (**b**) The rpart package in R was used, and 10-fold cross-validation process repeated 10 times to select the best fitting.

A RadScore was built using the decision-tree analysis, based on the radiomics parameters including mean ADC value of the tumor (ADCt_mean_: cutoff, 1.1 × 10^−3^ mm^2^/s), skewness of the relative ADC value (rADC_skewness_: cutoff, 1.2), short-axis diameter of LN (cutoff, 1.7 mm) and skewness ADC value of the LN (ADCln_skewness_: cutoff, 7.2 × 10^−2^) (Figure 3a). The characteristics of patients according to the risk group based on the radiomics parameters (RadScore) is detailed in Table 2. A RadSignature was composed based on the RadScore, tumor grade (1 and 2 vs. 3), and clinical tumor size (cutoff, 20 mm) (Figure 3b).

Bootstrap analysis of the training dataset revealed a sensitivity, specificity, accuracy, positive predictive value, and NPV of 95.51% (87.9–100%), 86.91% (82.8–90.9%), 87.78% (84.2–91.2%), 45.06% (38.3–53.4%), and 99.42% (98.4–100%), respectively, for the RadSignature on a regional basis. The ADC model was based solely on the mean ADC value of the LN (ADCln_mean_ < 1.1 × 10^−3^ mm^2^/s). The SA model was based on a short-axis diameter of LN > 5 mm.

The diagnostic performances of models for the detection of pelvic LN metastasis are summarized in Table 3. On the regional basis, the sensitivity of the RadSignature for detecting LN metastasis (100%) was significantly higher than that of the ADC (44%, *p* = 0.0001) or SA model (76%, *p* = 0.0313). The specificity of the RadSignature for detecting LN metastasis (91%) was also significantly higher than that of the ADC (75%, *p* < 0.0001) or SA model (61%, *p* < 0.0001), for the testing dataset. On a per patient basis, the sensitivity of the RadSignature for detecting LN metastasis (100%) was significantly higher than that of the ADC model (59%, *p* = 0.0156). The sensitivity of RadSignature was also higher than SA model (88%, *p* = 0.5), but did not reach statistical significance level. The specificity of the RadSignature to detect LN metastasis (90%) was significantly higher than that of the ADC (67%, *p* = 0.0001) or SA model (41%, *p* < 0.0001), for the testing dataset. Based on the ROC analysis, the RadSignature significantly outperformed the ADC and SA models for both the region and patient bases.

**Figure 4:**
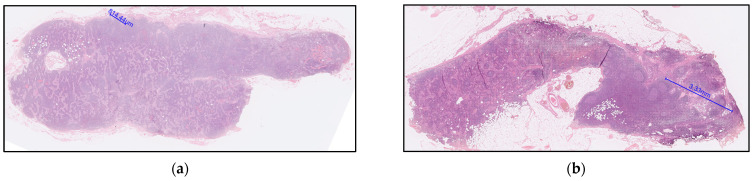
Explanation of the two false-negative cases based on the RadSignature. (**a**) A 40-year-old patient with grade 2 endometrioid adenocarcinoma with squamous differentiation, low segment and cervical stromal involvement, pathological staging T2N1mi; tumor size of 6.5 cm, and serum CA125 level of 37.3 U/mL. The lymph node (LN) was regarded false-negative based on the RadSignature criteria: mean ADC value of the tumor (ADCt_mean_: 0.928 × 10^−3^ mm^2^/s), skewness of the relative ADC value (rADC_skewness_: 0.051), short-axis diameter of LN (1.506 mm) and skewness ADC value of the LN (0.413 × 10^−2^). Microscopic nodal metastasis with a tumor nest size of 0.8 mm was identified based upon the hematoxylin and eosin (H&E) stain. (**b**) A 6-year-old patient with grade 2 endometrioid adenocarcinoma, no low segment and cervical stromal involvement, pathological staging T1bN1a; no low segment involvement, tumor size 1 cm, CA125 = 24.3 U/mL. The LN was regarded false-negative based on the RadSignature criteria: mean ADC value of the tumor (ADCt_mean_: 0.779 × 10^−3^ mm^2^/s), skewness of the relative ADC value (rADC_skewness_: 2.3263), short-axis diameter of LN (3.364 mm) and skewness ADC value of the LN (2.178 × 10^−2^). Nodal metastasis with a tumor nest size of 3.3 mm was identified based upon the H&E stain.

The pairwise comparisons of ROC curves in detecting pelvic lymph node metastasis is summarized in Table 4. The only two false-negativity of the RadSignature demonstrated microscopic tumor nests of 0.8 mm and 3.3 mm, respectively (Figure 4).

The implication of over-diagnosis causes an unnecessary LN dissection, particularly in low-risk patients. Therefore, we conducted a post-hoc subgroup analysis to investigate the possibility of the over-diagnosis or under-diagnosis in a specific risk group. Subgroups were defined according to the European Society of Gynaecological Oncology–European Society for Medical Oncology guidelines [24]: (1) low-risk (stage IA, grade 1–2, endometrioid type), (2) intermediate-risk (stage IA, grade 3 EC or IB grade 1–2 endometrioid type), (3) high-risk (Stage IB, grade 3 endometrioid type or any stage any grade non-endometrioid type). The RadSignature outperformed the ADC and SA models in all the risk groups for all the study participants (Table 5). Notably, on the per patient basis, the RadSignature retained a sensitivity of 100% to detect LN metastasis in all groups; moreover, its specificity was significantly higher than that of the ADC (*p* = 0.0005) or SA model (*p* < 0.0001) in the low-risk group. The specificity of the tree model to detect LN metastasis (86%) was significantly higher than that of the SA model in the intermediate-risk (*p* = 0.0078) and high-risk (*p* = 0.0313) groups.

## 4. Discussion

In the present study, we combined all available clinical and MR imaging parameters to build a composite prediction model— the RadSignature. The major advantages of decision-tree analysis are ease in interpretation of the tree using the binary splitting rule, which efficiently balances model accuracy and model simplicity or interpretability, and familiarity of the end user with the modeling technique. The RadSignature model yields an excellent NPV (98%), thus a subset of low-risk patients with EC who may not benefit from lymphadenectomy can be reliably identified. For patients undergoing lymphadenectomy, the prediction model could guide surgery through localization of potential laterality of nodal metastasis with reasonable accuracy. To the best of our knowledge, this is the first model predicting LN metastasis in EC based on the most comprehensive clinical and radiomic information obtained preoperatively.

Although not selected in the decision-tree model, we found that the ADC_mean_ and ADC_min_ of metastatic LNs were significantly lower than those of the benign LNs. Our findings are in line with a previous study that showed that the ADC_mean_ and ADC_min_ of metastatic LNs are significantly lower than those of non-metastatic LNs [10]. A recent publication supporting this point demonstrated that the ADC metrics of lymph nodes, including ADC_min_, ADC_max_, ADC_mean_, ADC_SD_, and rADC, showed high values enabling differentiation between metastatic and non-metastatic lymph nodes [25]. However, other studies have reported contradictory results of no significant difference in the mean ADC values between metastatic and non-metastatic nodes either at 1.5 T with *b* = 0, 800 mm^2^/s [9] or at 3 T with *b* = 0, 1000 mm^2^/s [8]. The controversial result might be attributed to potential bias in manual measurement which might be solved by using computer-aided segmentation in the present study.

LN short-axis diameter is indeed an outstanding factor [10,26] for predicting LN metastasis in EC and was selected in the present decision-tree analysis. However, a study reported no size differences between the metastatic and non-metastatic nodes on T2W images, but reported a significant difference on pathology slices [9]. Such controversial results imply the potential pitfall of LN segmentation on MR imaging, thereby again highlighting the computer-assisted segmentation technique applied in this study could reduce the potential bias caused by selecting small ROI of LN. Notably, based on our previous work, combining size and relative ADC values can result in higher sensitivity (25% vs. 83%) but similar specificity (98% vs. 99%) to detect LN in gynecologic cancers compared to conventional MR imaging [11], with the smallest detected metastatic LN being of 5 mm on its short axis [11].

In the present study, grade I EC with a tumor size < 20 mm can reliably exclude nodal metastasis, as supported by the results from previous studies [6,15,17]. Studies have also shown that preoperative assessment based on MR imaging and tumor histological grade can identify low-risk patients for nodal metastasis, and lymphadenectomy may be omitted in this subgroup of patients [17,27]. The Mayo-modified criteria (well or moderately differentiated endometrioid histology, <50% invasion, and tumor size < 20 mm) are also widely applied to assess nodal disease risk in patients with EC [28]. Our data and all the aforementioned models suggest tumor histology grade and size remain a central role of preoperative assessment for LN metastasis.

Our proof-of-concept model, although seemingly promising, has several limitations that merit further discussion. First, the overfitting of the model may occur due to the smaller sample size relative to the number of features extracted. Although the statistical power was sufficient, as well as the cross-validation and independent set being tested in this prospective study, our preliminary results must be validated externally before a wider adaptation into a clinical decision process. Second, the radiomic features extracted from the images are related to histogram analysis of ADC value while not including the higher order texture analysis, because 97% of the lymph node regions contain <100 pixels for analysis. Third, some imaging characteristics of the LNs (such as LN margin) were not included in the algorithm. Lobulated and spiculated LN margins indicate metastatic LNs, whereas smooth margin suggests benign LNs. Inclusion of this information might further enhance the performance of the prediction model. Finally, region-based analysis was used in this study, but we were unable to assess precise node-to-node radiological pathology correlation. Nevertheless, the strength of the present study is that the computer-assisted segmentation technique could reduce the potential bias caused by selecting small lymph nodes in pelvic MR. The decision-tree learning method has an advantage in interpretation using the binary splitting rule, which efficiently balances model accuracy and model simplicity or interpretability.

## 5. Conclusions

In conclusion, computer-aided segmentation and machine learning added values of clinical parameters and DW radiomics for predicting nodal metastasis in EC, with a diagnostic performance superior to that of the current ADC and size criteria. The high-throughput radiomic ADC features through machine learning have potential in building a prediction model to serve as a risk stratification tool for lymphadenectomy and guide the extent of operation through the localization of potential LN metastasis regions.

## Figures and Tables

**Table 1:** Demographics of the study participants.

Variables	RadScore			*p*-Value
	All	Negative	Positive	
*n*	236 (100.0)	213 (90.3)	23 (9.7)	
Age (year, mean ± SD)	51.2 ± 11.6	50.6 ± 11.8	56.2 ± 7.7	0.004 *
Histology				<0.0001 *
Non-endometrioid type	17 (7.2)	9 (3.8)	8 (3.4)	
Endometrioid type	219 (92.8)	204 (86.5)	15 (6.3)	
Grade				<0.0001 *
3	44 (18.6)	30 (12.7)	14 (5.9)	
1 + 2	192 (81.4)	183 (77.6)	9 (3.8)	
Tumor size ≥ 20 mm				0.009 *
Presence	140 (59.3)	120 (50.8)	20 (8.5)	
Absence	96 (40.7)	93 (39.5)	3 (1.2)	
Deep myometrial invasion				<0.0001 *
Presence	55 (23.3)	39 (16.6)	16 (6.7)	
Absence	181 (76.7)	174 (73.7)	7 (3.0)	
Low segment involvement				0.002 *
Presence	138 (58.5)	117 (49.6)	21 (8.9)	
Absence	98 (41.5)	96 (40.7)	2 (0.8)	
CA125 (mean ± SD, U/mL)	52.4 ± 202.4	33.2 ± 43.9	230.7 ± 618.2	0.140

SD = standard deviation. * *p* < 0.05, Data in parenthesis represents percentage.

**Table 2:** Characteristics of patients according to the risk group based on radiomics parameters (RadScore).

Variables	Lymph Node Metastasis			*p*-Value
	All	Absent	Present	
*n*	236 (100.0)	151 (64.0)	85 (36.0)	
Age (year, mean ± SD)	51.2 ± 11.6	49.8 ± 11.8	53.6 ± 10.8	0.017 *
Histology				0.005 *
Non-endometrioid type	17 (7.2)	5 (2.1)	12 (5.1)	
Endometrioid type	219 (92.8)	146 (61.9)	73 (30.9)	
Grade				0.049 *
3	44 (18.6)	22 (9.3)	22 (9.3)	
1 + 2	192 (81.4)	129 (54.7)	63 (26.7)	
Tumor size ≥ 20 mm				0.002 *
Presence	140 (59.3)	78 (33.1)	62 (26.2)	
Absence	96 (40.7)	73 (30.9)	23 (9.8)	
Deep myometrial invasion				<0.0001 *
Presence	55 (23.3)	20 (8.5)	35 (14.8)	
Absence	181 (76.7)	131 (55.5)	50 (21.2)	
Low segment involvement				0.015 *
Presence	138 (58.5)	79 (33.5)	59 (25.0)	
Absence	98 (41.5)	72 (30.5)	26 (11.0)	
CA125 (mean ± SD, U/mL)	52.4 ± 202.4	32.4 ± 36.3	87.9 ± 332.0	0.129

SD = standard deviation. * *p* < 0.05, Data in parenthesis represents percentage.

**Table 3:** Diagnostic accuracy for detecting pelvic lymph node metastasis based on selected magnetic resonance (MR) imaging features.

Parameters	*n*	TP	TN	FP	FN	Sensitivity	Specificity	Accuracy
Region basis (Training)								
RadSignature	330	22	267	40	1	95.7% (78.1–99.9%)	87.0% (82.7–90.5%)	87.6% (83.5–90.9%)
RadScore	330	22	248	59	1	95.7% (78.1–99.9%)	80.8% (75.9–85.0%)	81.8% (77.2–85.8%)
ADC	330	21	91	216	2	91.3% (72.0–98.9%)	29.6% (24.6–35.1%) *	33.9% (28.8–39.3%) *
SA	330	16	213	94	7	69.6% (47.1–86.8%) *	69.4% (63.9–74.5%) *	69.4% (64.1–74.3%) *
Region basis (Testing)								
RadSignature	142	8	114	18	2	80.0% (44.4–97.5%)	86.4% (79.3–91.7%)	85.9% (79.1–91.2%)
RadScore	142	8	106	26	2	80.0% (44.4–97.5%)	80.3% (72.5–86.7%)	80.3% (72.8–86.5%)
ADC	142	7	32	100	3	70.0% (34.8–93.3%)	24.2% (17.2–32.5%) *	27.5% (20.3–35.6%) *
SA	142	7	85	47	3	70.0% (34.8–93.3%)	64.4% (55.6–72.5%) *	64.8% (56.3–72.6%) *
Patient basis (Training)								
RadSignature	165	15	119	30	1	93.8% (69.8–99.8%)	79.9% (72.5–86.0%)	81.2% (74.4–86.9%)
RadScore	165	15	105	44	1	93.8% (69.8–99.8%)	70.5% (62.5–77.7%)	72.7% (65.3–79.4%)
ADC	165	15	25	124	1	93.8% (69.8–99.8%)	16.8% (11.2–23.8%) *	24.2% (17.9–31.5%) *
SA	165	12	80	69	4	75.0% (47.6–92.7%)	53.7% (45.3–61.9%) *	55.8% (47.8–63.5%) *
Patient basis (Testing)								
RadSignature	71	6	50	14	1	85.7% (42.1–99.6%)	78.1% (66.0–87.5%)	78.9% (67.6–87.7%)
RadScore	71	6	44	20	1	85.7% (42.1–99.6%)	68.8% (55.9–79.8%)	70.4% (58.4–80.7%)
ADC	71	6	9	55	1	85.7% (42.1–99.6%)	14.1% (6.6–25.0%) *	21.1% (12.3–32.4%) *
SA	71	5	28	36	2	71.4% (29.0–96.3%)	43.8% (31.4–56.7%) *	46.5% (34.5–58.7%) *

Data in parentheses are 95% confidence intervals. TP = true positive, TN = true negative, FP = false positive, FN = false negative, PPV = positive predictive value, NPV = negative predictive value, SA = short axis, ADC = mean apparent diffusion coefficient value of the lymph node. * *p* < 0.05 McNemar test, as compared with the RadSignature model.

**Table 4:** Pairwise comparisons of ROC curves in detecting pelvic lymph node metastasis.

Variables	AUC Value (95% CI)	*p* Value
Region basis		
RadSignature	0.89 (0.86–0.92)	
ADC	0.56 (0.52–0.61)	
SA	0.69 (0.64–0.73)	
RadSignature vs. ADC		<0.0001 ^†^
RadSignature vs. SA		<0.0001 ^†^
ADC vs. SA		0.0406
Patient basis		
RadSignature	0.85 (0.80–0.90)	
ADC	0.54 (0.47–0.60)	
SA	0.62 (0.56–0.69)	
RadSignature vs. ADC		<0.0001 ^†^
RadSignature vs. SA		<0.0001 ^†^
ADC vs. SA		0.1742

Data in parentheses are 95% confidence intervals. ROC = receiver operating characteristic, SA = short axis, ADC = mean apparent diffusion coefficient value of the lymph node. † The differences were significant according to Bonferroni correction for multiple comparisons.

**Table 5:** Subgroup analysis of diagnostic accuracy of MR imaging for detecting pelvic lymph node metastasis.

Parameters	*n*	TP	TN	FP	FN	Sensitivity	Specificity	Accuracy
Low risk								
RadSignature	165	4	135	25	1	80.0% (28.4–99.5%)	84.4% (77.8–89.6%)	84.2% (77.8–89.4%)
RadScore	165	4	116	44	1	80.0% (28.4–99.5%)	72.5% (64.9–79.3%)	72.7% (65.3–79.4%)
ADC	165	5	26	134	0	100.0% (47.8–100.0%)	16.3% (10.9–22.9%) *	18.8% (13.1–25.6%) *
SA	165	1	86	74	4	20.0% (0.5–71.6%) *	53.8% (45.7–61.7%) *	52.7% (44.8–60.5%) *
Intermediate risk								
RadSignature	36	3	20	12	1	75.0% (19.4–99.4%)	62.5% (43.7–78.9%)	63.9% (46.2–79.2%)
RadScore	36	3	19	13	1	75.0% (19.4–99.4%)	59.4% (40.6–76.3%)	61.1% (43.5–76.9%)
ADC	36	4	4	28	0	100.0% (39.8–100.0%)	12.5% (3.5–29.0%) *	22.2% (10.1–39.2%) *
SA	36	2	14	18	2	50.0% (6.8–93.2%)	43.8% (26.4–62.3%) *	44.4% (27.9–61.9%) *
High risk								
RadSignature	35	14	14	7	0	100.0% (76.8–100.0%)	66.7% (43.0–85.4%)	80.0% (63.1–91.6%)
RadScore	35	14	14	7	0	100.0% (76.8–100.0%)	66.7% (43.0–85.4%)	80.0% (63.1–91.6%)
ADC	35	12	4	17	2	85.7% (57.2–98.2%)	19.0% (5.4–41.9%) *	45.7% (28.8–63.4%) *
SA	35	14	8	13	0	100.0% (76.8–100.0%)	38.1% (18.1–61.6%) *	62.9% (44.9–78.5%) *

Data in parentheses are 95% confidence intervals. TP = true positive, TN = true negative, FP = false positive, FN = false negative, PPV = positive predictive value, NPV = negative predictive value, SA = short axis, ADC = mean apparent diffusion coefficient value of the lymph node. * *p* < 0.05 McNemar test, as compared with the RadSignature model.

## Data Availability

Data of this study will be available upon request.

## Appendix A

### Appendix A.1. Imaging Protocol

All exams were conducted with a 3-T MR scanner (Tim Trio; Siemens, Erlangen, Germany) before the patients were scheduled for operations. The integrated spine coil and body-phased array coil were used to cover the entire pelvis. Pelvic T2-weighted (T2W) and DW imaging were acquired with identical slice thicknesses and gaps in the true sagittal and axial planes. High-resolution T2W imaging was performed using fast spin-echo sequences (repetition time [TR] ms/echo time [TE] ms, 5630/87; average, 3; matrix, 256 × 320; field of view [FOV], 20 cm). DW imaging was performed using a single-shot echo-planar technique with fat suppression (TR/TE, 3300/79; average, 4; slice thickness, 4 mm; gap, 1 mm; matrix, 128 × 128; FOV, 20 cm). The MR examinations were conducted during minimal breathing. No premedication was administered. ADCs were calculated using the slope of logarithmic monoexponential signal intensity decay curve against the *b* values of 0 and 1000 s/mm^2^ (VB17a, Syngo; Siemens).

### Appendix A.2. Segmentation of Tumors and Lymph Nodes (LNs)

The anonymous magnetic resonance imaging (MRI) dataset was digitally transferred from the picture archiving and communication system workstation (GE Centricity PACS RA1000; GE Healthcare, Milwaukee, WI, USA) to a personal computer with an Intel Core i7-5960X 2.4-GHz processor and 3 GB RAM running Windows 7 in a 64-bit environment. By using an in-house developed software written in MATLAB version 8.3 R2014a (MathWorks, Natick, MA, USA), the first reader (Y.T.H, a gynecologic radiologist with eight years of experience) drew regions of interest (ROIs) around the tumor on each section on the high *b*-value diffusion weighted images (DWIs) with reference to the apparent diffusion coefficient (ADC) maps and T2-weighted images. The second reader independently verified the ROIs (G.L, a gynecologic radiologist with 10 years of experience). Both readers were blinded to clinical outcome. Care was taken to avoid ROI contaminated by the adjacent normal cervical stroma or vascular structures, or by areas of fluid or Nabothian cysts in the cervix. Normalized ADC (nADC) was computed and used for comparison in this study. The nADC was defined as ADC (tumor or LN)/ADC (reference). ADC reference value was obtained from the urine, with ovoid ROI placed in the center of the bladder lumen. Due to the small size of pelvic LNs, which made them difficult for accurate manual segmentation by MRI, an application software with a graphic user interface (Figure 2) specific for LN segmentation developed in-house was used for this purpose. The largest LNs in the left and right pelvic areas were identified on axial pelvic DWI along with T2-weighted images (T.Y.S, a radiologist with two years of experience). The identified LN was automatically segmented using a region-growing algorithm (https://www.mathworks.com/matlabcentral/fileexchange/19084-region-growing/content/regiongrowing.m; accessed on 7 February 2020). Specifically, a pixel is initially selected as the starting point of a region. The region is grown iteratively by comparing the neighboring pixels to the region. In every iteration, the neighboring pixel with the smallest intensity difference to the region mean is included to the region. This process terminates when the intensity difference between region mean and all neighboring pixels exceed a predefined threshold, thereby providing a 100% reproducibility of segmentation. The reason we use 2D instead of 3D is that LN size is too small to be segmented in 3D.

### Appendix A.3. Surgical Procedure and Histopathology

Primary surgical treatment consisted of hysterectomy, bilateral salpingo-oophorectomy and pelvic LN dissection. Para-aortic LN dissection was carried out for patients with high-risk histopathological type or with clinical suspicion of deep myometrial invasion. The resected nodes were anatomically labeled in left or right pelvic regions by the surgeons. The LNs were then cut into parallel slices of thickness 2 to 3 mm. All nodal tissue was routinely processed and embedded in paraffin, followed by staining with hematoxylin and eosin. The histopathology report included the number of total harvested LNs and the identified metastatic ones in each region respectively. Histopathologic types and tumor grades were evaluated in the consensus of a general pathologist and a specialized gynecological pathologist (R.C.W), with relevant clinical information available. A consensus read between surgeons, pathologists and radiologists for providing the most accurate assignment between MR and histopathology were performed in the weekly panel discussion of gynecologic oncology.

### Appendix A.4. Statistical Analysis

The prediction problem was set to predict pathological LN metastasis, and the target variable was categorical (presence or absence). The decision tree used in this study used iterative back propagation, but unlike deep learning, the decision tree can identify important parameters and determine the priority of the steps to construct an actionable plan. A decision tree, in general, does not have the best predictive accuracy compared with some other machine learning techniques. However, it has the advantage of interpretability, with a format consistent with many clinical pathways. A decision tree based on classification and regression tree method was used to identify combined clinical and MR parameters predictive for LN metastasis. The tree construction was performed using R package rpart (R Foundation, http://cran.r-project.org/web/packages/rpart/rpart.pdf; accessed on 8 August 2020). The classification rules are applied sequentially with each rule partitioning a predictor (so-called attribute) into a binary response. The splitting rule was built based on minimizing the impurity in the attribute, so that selecting a root attribute could vary according to the splitting rule and scaling. The classification and regression tree method automatically identified and removed redundant independent variables. The practical costs of prediction errors would be more important to prevent underdiagnosis. In order to learn a model which have high sensitivity and acceptable accuracy and specificity, based on the data with imbalance structure in LN positive rate, a weighted algorithm was introduced. The selected weight value led to the achievement of 90% sensitivity. The success criteria for prediction were set to achieve high sensitivity and NPV whilst remaining non-inferior specificity to the standard of care, based on metrics in internal validation. The sample size and power calculation of machine learning methods need to be constructed by numerical simulation and estimation. In general, the power can achieve more than 90% if sample size per class is more than 4 at significance level 5%, for a number of groups being less than 10 [29]. In the present study, we optimized the growing of the tree, setting minsplit = 5 and maxdepth = 4, to control the size to avoid over-fitting and low-power. We did not perform data preprocessing, including data cleaning or transformation or outlier removal. Missing data were excluded from the analysis case-wise.
